# Phenotyping and genotyping studies on extended-spectrum β-lactamase-producing *Escherichia coli* isolates from mastitic cows on dairy farms in Egypt

**DOI:** 10.14202/vetworld.2022.890-897

**Published:** 2022-04-11

**Authors:** Shereen S. El-Mohandes, Rasha H. Eid, Ahmad M. Allam, Hala A. A. Abou-Zeina, Mohamed K. Elbayoumy

**Affiliations:** 1 Department of Mastitis and Neonatal Diseases, Animal Reproduction Research Institute, Agriculture Research Center, Giza 12622, Egypt; 2 Department of Parasitology and Animal Diseases, Veterinary Research Institute, National Research Centre, Giza 12556, Egypt

**Keywords:** antibiotic resistance, *Escherichia coli*, extended-spectrum β-lactamases, mastitis

## Abstract

**Background and Aim:**

Extended-spectrum β-lactamase (ESBL)-producing Enterobacteriaceae have become a serious public health hazard worldwide. This importance is derived from the increase of new variants, particularly *blaTEM*, *blaSHV*, and *blaCTX-M* genes. This study aimed to examine ESBL-producing *Escherichia coli* isolated from different governorates in Egypt from dairy cows infected with subclinical and clinical mastitis.

**Materials and Methods:**

This study examined 207 milk samples for the resistance of isolates against 14 different antibiotics and ran serological identification of ESBL-producing *E. coli* isolates with complete antibiotic resistance. Genotypic and sequencing analyses of several resistance genes were conducted using a polymerase chain reaction.

**Results:**

*E. coli* was identified in cases with subclinical mastitis (80.5%) and clinical mastitis (85.7%). ESBL-producing *E. coli* was isolated from 38.2% of subclinical mastitic milk compared to 39.3% in clinical cases, where O26:k60, O125:k70, and O25:k11 were the serotypes with complete resistance to antibiotics. ESBL-producing *E. coli* isolates were resistant to cefotaxime, amoxicillin, cloxacillin, oxacillin, rifampicin, and penicillin in 100% but susceptible to amoxicillin and clavulanic acid in 82.5% of the cases. Results also revealed that 51.25%, 52.5%, 66.25%, 77.5% and 60% of ESBL-producing *E. coli* isolates were responsive to ciprofloxacin, ofloxacin, norfloxacin, levofloxacin, and gentamycin, respectively. The detected genes were registered in GenBank as MW345819.1 and MW345820.1 for the *E. coli* *blaTEM* gene and MW295407 for the *E. coli* *blaSHV* gene.

**Conclusion:**

This study found ESBL-producing *E. coli* in mastitic milk samples from Egyptian dairy farms and confirmed the occurrence and circulation of the main antibiotic genes (*blaTEM* and *blaSHV*) in the samples. Regular and thorough surveillance of ESBL-producing *E. coli* and subsequent preventive actions are essential for preventing the spread of these resistance genes in the future, which could pose serious and catastrophic health risks. Authorities should cling to the concept of One Health to minimize the risk of new varieties.

## Introduction

Mastitis is one of the most important and dangerous infectious diseases in the dairy sector, as it poses a danger to human health and affects animal welfare. Mastitis can be caused by various bacteria, with *Escherichia coli* being one of the most common [1]. *E. coli* is a Gram-negative bacterium present as commensals in humans and animals and can acquire and retain transferable resistance genes [2].

Multidrug resistance (MDR) is a multifaceted public health issue that involves humans and animals. One of the influential resistance mechanisms in *E. coli* that decrease the efficacy of β-lactam antibiotics is based on the plasmid-mediated production of enzymes that inactivate these compounds by hydrolyzing their β-lactam ring. These variants are called extended-spectrum β-lactamases (ESBL) [3], leading to antimicrobial resistance in bacteria, and emerging as a problem in both human and veterinary medicine [4]. A particular concern has been seriously observed in ESBL producers, increasing worldwide threats against public healthcare. It is associated with other commonly used antibiotics, such as aminoglycosides, fluoroquinolones, and tetracyclines [5].

A wide variety of β-lactam antibiotics are commonly used to treat, control, and prevent many infectious conditions of Gram-negative bacteria from bovine mastitis isolates. In particular, the antibiotic resistance caused by these bacteria leads to increased morbidity, mortality rates, and treatment cost [6]. In human patients with bacteremia of the urinary and biliary tracts, the severity of ESBL-producing *E. coli* mortality is thrice higher than non-ESBL-producing *E. coli* [7]. ESBL-producing *E. coli* are MDR independent from the source and show resistance to cephalosporins and β-lactam and non-β-lactam antibiotics. Importantly, ESBL genes may outspread by clonal diffusion of the host or through mobile genetic elements [8], indicating that antimicrobial resistance genes and/or host bacteria may be exchanged between animals and humans, imposing a coordinated health policy to manage it. Food animals, such as cattle, are reservoirs for Enterobacteriaceae that produce ESBLs and can be transferred through the food chain and animal contact [8,9].

Enterobacteriaceae that produce this type of resistance has become of public health significance worldwide since 1995 due to the emergence of new variants, such as TEM genes (*blaTEM*), SHV genes (*blaSHV*), and CTX-M genes (*blaCTX-M*) [10]. This continued evolution is a serious hazard to public health, diminishing the treatment power of antimicrobials [11,12].

In the past two decades, studies recorded the dissemination of ESBL-producing *E. coli* in food-producing animals in several countries, such as in Europe [13] and Asia [14]. In dairy farms, raw milk from mastitic cows is considered a potential contributor to ESBL-producing *E. coli* and their encoding genes [15]. The ESBL-encoding genetic elements are transferable between the same and different bacterial species [16].

This study aimed to investigate the antibiotic susceptibility, serological identification, genotyping, and sequencing of these genes in ESBL-producing *E. coli* isolated from dairy cattle with subclinical and clinical mastitis in four Egyptian governorates (Giza, Menofia, Beheira, and Alexandria).

## Materials and Methods

### Ethical approval

In conformity with local laws and regulations, the Ethical Committee for Medical Research of Egypt’s National Research Centre (NRC) authorized this study under the number 16229.

### Study period and location

The study was conducted from August 1, 2020, to August 20, 2021. The samples were collected from four Egyptian governorates (Giza, Menofia, Beheira, and Alexandria). The samples were analyzed at Animal Reproduction Research Institute and National Research Centre, Giza, Egypt.

### Collection of milk samples

**Figure-1:**
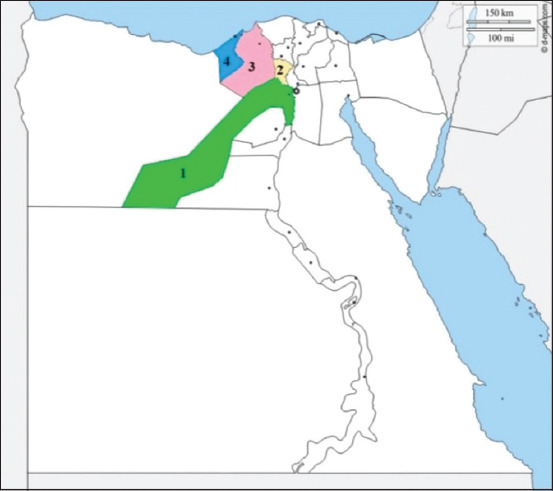
Map of Egypt showing governorates where milk samples were collected: 1-Giza, 2-Menofia, 3-Beheira, 4-Alexandria [Source: https://d-maps.com/m/africa/egypte/egypte76.gif].

Four commercial dairy herds located in various Egyptian governorates (Giza, Menofia, Beheira, and Alexandria) mentioned in Figure-1, with a medium-scale herd size ranging from 60 to 160 dairy cattle, were investigated. The cattle in this study ranged from 2 to 10 years old. Clinical examination of these animals was performed to detect any clinical abnormalities, with special emphasis on the udder by visual inspection and palpation to diagnose clinical mastitis. The California mastitis test was used to detect subclinical mastitis [17]. This study included 207 milk samples from the four farms (123 subclinical cases and 84 clinical cases). After disinfecting the teat with 70% alcohol, 25-mL milk was collected into sterile screw-capped bottles under appropriate hygienic conditions from each quarter. Each quarter’s first three squirts were discarded. Within 24 h, the milk samples were maintained on ice and transported to the laboratory. All samples were kept at 4°C from when they were collected until they were examined by conventional techniques within 3-4 h for bacteriological examination [18].

### Isolation of *E. coli*

The milk samples were incubated for 18 to 24 h at 37°C, and a loopful from the samples was cultured on MacConkey agar and blood agar medium. All plates were incubated at 37°C for 24-48 h and subjected to isolation and identification of *E. coli*. Pink lactose-fermenting colonies of ≥3 mm were identified as suspected colonies of *E. coli*. All bacterial isolates were biochemically validated using (indole, methyl red, Voges-Proskauer, and citrate tests, and triple sugar iron) for the isolation and confirmation of *E. coli* strains. Films from the suspected colonies were prepared and stained with Gram stain. *E. coli* appeared as Gram-negative rods, smooth, nonsporulated, and medium-sized. *E. coli* isolates were transferred to 20% glycerol for further identification according to Quinn [19]. For phenotypic identification of ESBL-producing *E. coli*, 1 mg/L cefotaxime (CFX) was added to MacConkey agar. This medium can differentiate ESBL-positive and ESBL-negative strains; CFX inhibits non-ESBL-producing *E. coli* but allows ESBL strains to grow [20].

### Phenotypic identification of ESBL-producing *E. coli*

*E. coli* isolates were first screened for the phenotypic identification of ESBL producers by culturing on selective MacConkey agar plates supplemented with 1-mg/L CFX, and then ESBL-producing *E. coli* were grown [20].

### Antibiotic susceptibility testing

Antibiotic susceptibility of ESBL-producing *E. coli* isolates was carried out on Mueller-Hinton agar (HiMedia, Mumbai, India) against 14 different commercially available antibiotics disks (CFX [30 μg] ciprofloxacin [CIP] [5 μg], norfloxacin [NOR] [10 μg], ofloxacin [OFX**]** [5 μg], levofloxacin [LEV] [5 μg] amoxicillin-clavulanic acid [AMC] [30 μg], amoxicillin [AMX] [25 μg], penicillin [P] [10 U], neomycin [30 μg], cloxacillin [CX**]** [1 μg], oxacillin [OX**]** [1 μg], gentamycin [GN] [10 μg], cefquinome [30 μg], and Rifampicin [RD] [5 μg] using the disk diffusion technique. Susceptibility was recorded by measuring the inhibition zones and scored as sensitive and resistant according to the recommendations of [21].

### Serological identification

Serological identification was performed on ESBL-producing *E. coli* isolates according to the manufacturer’s manual (anti-coli Sifin diagnostic kits GmbH, Berlin, Germany) used for O-serogrouping [22].

### Deoxyribonucleic acid (DNA) extraction

DNA was extracted from bacterial colonies using a Nucleic Acid Extraction Kit (Vivantis [GF-1] Malaysia) according to the manufacturer’s protocol. Briefly, bacterial colonies were collected from the agar medium, and 20-mL lysozyme was added to the bacterial suspension and mixed thoroughly. After centrifugation, the resuspended pellets were treated with proteinase K at 65°C with occasional shaking. A homogenization step by adding absolute ethanol was applied. The treated solution was added to the spinning column and washed and dried by centrifugation. DNA elusion was performed using 50-μL elusion buffer. The genomic DNA concentrations were assessed using a spectrophotometer (Nanodrop Micro volume spectrophotometer Quawell Q9000CM), and the DNA samples were kept at 20°C until polymerase chain reaction (PCR).

### Screening of ESBL-producing *E. coli* DNA using standard PCR

The primer sets of the genes encoding ESBL production (*blaCTX-M*, *blaTEM*, *blaSHV*, and *blaCMY-2-group*) were selected according to Weiner *et al*. [23] as described in Table-1. PCR assay was run (BIO-RAD, Singapore) using One PCR Master Mix™ (GeneDireX, Taiwan). PCR was initiated by 5 min denaturation at 95°C, followed by 30 cycles consisting of denaturation at 95°C for 30 s, annealing at the appropriate temperature for each primer for 30 s, and extension for 30 s at 72°C. The last extension step was prolonged to 10 min. Amplification was confirmed by electrophoresis on a 1.5% agarose gel, stained with Red Safe (Intron, China), and examined by an ultraviolet transilluminator. A DNA molecular weight marker (100bp DNA Ladder H3 RTU, GeneDireX, Taiwan) was used to estimate the size of the products.

**Table 1:** Sequences of the primers used in ESBL *E. coli* identification.

Sequence (5×→3×)	Target gene	Annealing temperature °C	Amplicon
F: ATGTGCAGYACCAGTAARGTKATGGC R: TGGGTRAARTARGTSACCAGAAYCAGCGG	blaCTX	60	593 bp
F: TGAGTATTCAACATTTCCGTGT R: TTACCAATGCTTAATCAGTGA	blaTEM	53	861 bp
F: CAAAACGCCGGGTTATTC R: TTAGCGTTGCCAGTGCT	blaSHV	53	937 bp
F: GCACTTAGCCACCTATACGGCAG R: GCTTTTCAAGAATGCGCCAGG	blaCMY-2-group	60	758 bp

ESBL=Extended-spectrum β-lactamase, *E. coli*=*Escherichia coli*

### Sequencing and phylogenetic analyses

The purified PCR products were sequenced at Macrogen Lab Technology (Korea). The obtained sequences were assembled using Chromas Pro 1.7 (Technelysium Pty Ltd., Australia) and compared to GenBank (National Center for Biotechnology Information) using the Basic Local Alignment Search Tool (http://blast.ncbi.nlm.nih.gov/Blast.cgi). The maximum-likelihood phylogenetic trees were constructed using MEGAX [24] with 500 bootstrap replications.

## Results

After phenotypic identification, the percentages of *E. coli*-negative, ESBL-producing *E. coli*, and non-ESBL-producing *E. coli* were determined in 207 analyzed mastitic milk samples (Tables-2 and 3). *E. coli* were isolated from 80.48% and 85.71% of subclinical and clinical mastitis cases, respectively, whereas ESBL-producing *E. coli* were isolated from 38.2% and 39.3% of subclinical and clinical mastitis cases, respectively.

**Table 2:** Incidence of *E. coli* in examined mastitic milk samples.

Bacteria	Subclinical Mastitic milk samples (123)		Clinical Mastitic milk samples (84)		Total milk samples (207)	
		
	No.	%	No.	%	No.	%
*E. coli* isolates	99	80.5	72	85.7	171	82.6
Negative	24	19.5	12	14.3	36	17.4

Percentage was calculated according to the total number of each group. *E. coli*=*Escherichia coli*

**Table 3:** Incidence of ESBL - producing *E. coli* after Phenotypic identification.

*E. coli* isolates	Subclinical mastitic milk samples (123)		Clinical mastitic milk samples (84)		Total milk samples (207)	
		
	No.	%	No.	%	No.	%
ESBL *E. coli*	47	38.2	33	39.3	80	38.64
Non ESBL *E. coli*	52	42.3	39	46.4	91	43.96

Percentage was calculated according to the total number of each group. ESBL=Extended-spectrum β-lactamase,

*E. coli*=*Escherichia coli*

### Antibiotic susceptibility testing

**Figure-2:**
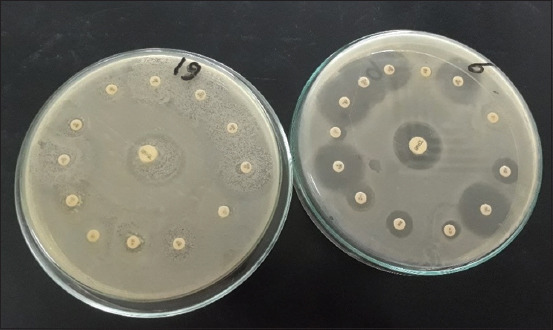
Antibiotic sensitivity test plates show complete and partial resistance of ESBL *E. coli*. ESBL=Extended-spectrum β-lactamase, *E. coli*=*Escherichia coli*.

Figure-2 shows the total and partial resistance of ESBL-producing *E. coli* on antibiotic sensitivity test plates. Table-4 shows the susceptibility pattern of 80 ESBL-producing *E. coli* isolates against 14 antimicrobial drugs. Regarding antibiotic sensitivities, isolates of ESBL-producing *E. coli* were resistant to CFX, AMX, CX, OX, RD, and P at 100% but sensitive to AMC 82.5%. Results also showed that ESBL-producing *E. coli* isolates were sensitive to CIP, OFX, NOR, LEV, and GN at 51.25%, 52.5%, 66.25%, 77.5%, and 60%, respectively.

**Table 4:** Antibiotic sensitivity against ESBL *E. coli.*

Antibiotics disks	ESBL*E. coli*(80)			

	Sensitive		Resistant	
	
	No.	%	No.	%
Cefotaxime	0	0	80	100
Ciprofloxacin	41	51.25	39	48.75
Ofloxacin	42	52.5	38	47.4
Norfloxacin	53	66.25	27	33.75
Levofloxacin	62	77.5	18	22.5
Amoxicillin-clavulanic acid	66	82.5	14	17.5
Amoxicillin	0	0	80	100
Neomycin	24	30	56	70
Cloxacillin	0	0	80	100
Oxacillin	0	0	80	100
Gentamycin	38	60	32	40
Cefquinome	16	20	64	80
Rifampicin	0	0	80	100
Penicillin	0	0	80	100

ESBL=Extended-spectrum β-lactamase,

*E. coli*=*Escherichia coli*

## PCR results

**Figure-3:**
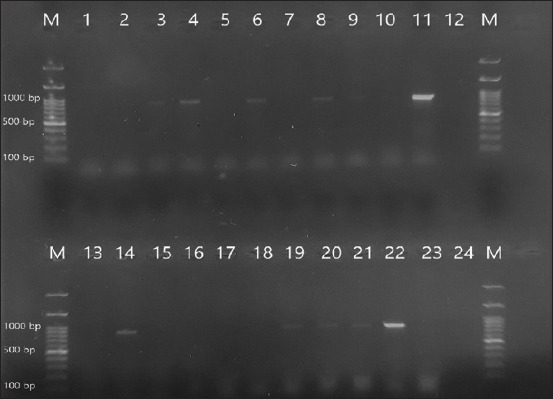
M: marker, Lanes (1, 12, 13 and 24): Negative control, Lanes (3, 4, 6, 8, 9, 11, 14, 19, 20, 21 and 22): Positive amplification of “blaTEM” DNA gene at 861 bp, Lanes (2, 5, 7, 10, 15, 16, 17 and 18): Negative amplification of “*blaTEM*” DNA gene. DNA=Deoxyribonucleic acid.

**Figure-4:**
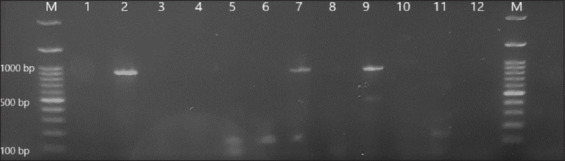
M: Marker, Lanes (1 and 12): Negative control, Lanes (2, 7 and 9): Positive amplification of “blaSHV” DNA gene at 937 bp, Lanes (3, 4, 5, 6, 8, 10 and 11): Negative amplification of “blaSHV” DNA gene. DNA=Deoxyribonucleic acid.

All 80 strains in this study were identified as *E. coli*. Based on growth on MacConkey agar and antibiotic susceptibility test results, these strained were evaluated as ESBL positive. The prevalence of different resistance genes *blaCTX-M*, *blaTEM*, *blaSHV*, and *blaCMY-2-group* in ESBL-producing *E. coli* isolates is presented in Table-5. PCR analysis is illustrated in Figures-3 and 4 for the isolated *blaTEM* and *blaSHV* genes. The *blaCTX* and *blaCMY-2-group* genes were not detected, whereas *blaTEM* was the most frequently isolated gene, followed by *blaSHV*, with frequency rates of 60% and 15%, respectively (Table-5).

**Table 5:** Prevalence of different resistant genes in ESBL *E. coli* isolates.

Type of resistant gene	Positive isolates (80)	

	No.	%
blaTEM	48	60
blaSHV	12	15
blaCTX	0	0
blaCMY-2-group	0	0

ESBL=Extended-spectrum β-lactamase,

*E. coli*=*Escherichia coli*

**Figure-5:**
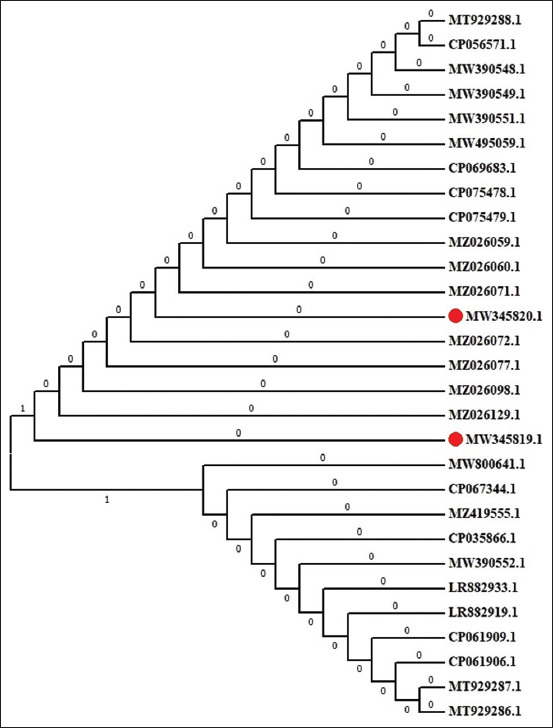
A maximum-likelihood phylogenetic tree highlighting the position of ESBL producing *E. coli* “blaTEM” nucleotide sequences genes (highlighted) related to other *E. coli* spp. available in GenBank. ESBL=Extended-spectrum β-lactamase, *E. coli*=*Escherichia coli*.

**Figure-6:**
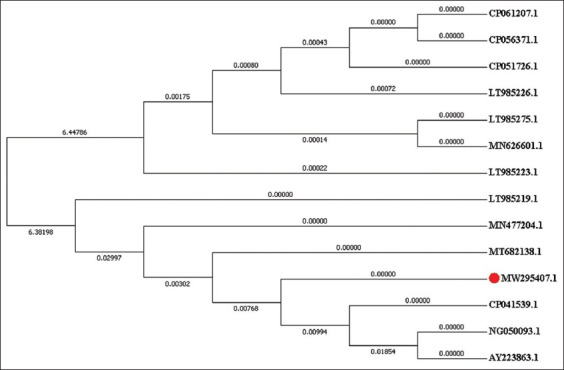
A maximum-likelihood phylogenetic tree highlighting the position of ESBL producing *E. coli* “blaSHV” nucleotide sequences genes (highlighted) related to other *E. coli* spp. available in GenBank. ESBL=Extended-spectrum β-lactamase, *E. coli*=*Escherichia coli*.

Figures-5 and 6 show a maximum-likelihood phylogenetic tree illustrating the position of isolated ESBL-producing *E. coli blaTEM* and *blaSHV* nucleotide sequence genes in relation to other *E. coli* spp. available in GenBank. The GenBank accession numbers in bankit for nucleotide sequences are MW345819.1 and MW345820.1 (Figure-5) for the *E. coli* TEM β-lactamase *blaTEM* gene and MW295407 (Figure-6) for the *E. coli* SHV β-lactamase *blaSHV* gene.

### Serological identification of ESBL-producing E. coli carrying β-lactam resistance genes

ESBL-producing *E. coli* isolates showed complete resistance to antibiotics. Those carrying *blaTEM* and *blaSHV* resistance genes produced the O26:k60, O125:k70, and O25:k11 serotypes.

## Discussion

Mastitis is a significant infection in dairy cows and an important problem for dairy farmers and the dairy industry. A wide range of bacteria are incriminated in producing the problem; nevertheless, the most common and serious bacterium is *E. coli*, an environmental pathogen inducing mastitis in dairy cows [25]. Signs of coliform mastitis can vary from mild inflammation in the quarter, minor alterations in milk appearance with no systemic signs, to severe clinical signs and a noticeable drop in milk production [26]. According to estimates, clinical mastitis accounts for 33%-38% of health costs for dairy herds. Mastitis is by far the most common reason for antibiotic usage in lactating dairy cattle, accounting for one-third of all antibiotics given to animals [27].

In the last decades, several studies showed that the production animals could be a reservoir and vehicle for the transmission and dissemination of ESBL due to their direct connection with the food chain of animal origin when these foods are directly consumed without undergoing any thermal process or used for raw milk [9,28].

This study found that *E. coli* was isolated from cases infected with both subclinical and clinical mastitis at 80.5% and 85.7%, respectively (Table-2). Similar results were obtained previously in France [29], showing that *E. coli* was isolated from >80% of cases of coliform mastitis. In contrast, in Egypt, the lower percentages were obtained by Ahmed *et al*. [30]. *E. coli* were isolated from 9.1% of clinical milk samples and 40% of subclinical mastitic milk samples. In another study in Egypt, *E. coli* were isolated from subclinical and clinical mastitis at 14% and 31%, respectively [31].

In addition, Table-3 clarifies incidences of ESBL-producing *E. coli* isolated from 38.2% of subclinical mastitic milk, whereas isolation was 39.3% in clinical cases. Nearly similar findings were obtained in Turkey [32], stating that the percentage of ESBL-*E. coli* grew from 33.2% in 2008 to 48.83% in 2013. Furthermore, in China [33], 23.53% of *E. coli* isolates from mastitic milk were proven to ESBL producers. A study conducted in Malaysia [34] showed that ESBL-producing *E. col*i were isolated from milk at a percentage of 66.7%. In Egypt and Germany, the incidence of ESBL-producing *E. coli* associated with cattle mastitis was 17% and 4.5%, respectively [31,35].

In general, the prevalence of ESBLs increases in different parts of the world. This could be explained and elucidated by the fact that resistance genes are frequently carried on plasmids that can be transferred from strain to strain and between bacterial species, increasing their prevalence. Some ESBL genes (e.g., *blaTEM/SHV*) are mutant derivatives of known plasmid-mediated β-lactamases, whereas others (e.g., *blaCTX-M*) are generated from environmental bacteria [36,37].

In terms of antibiotic sensitivities, the resistance patterns of bacterial populations differ between countries due to antimicrobial treatment disparities. For example, this study was almost identical to Batabyal *et al*. [38], who reported that the antibiogram of ESBL-positive isolates revealed drugs such as LEV (83.33%) to be highly sensitive against this pathogen but drugs such as CFX (100%) and gentamicin (58.33%) to be highly resistant. Furthermore, the resistance was 22% to GN and 15% to NOR [31]. Recently, all *E. coli* isolates were resistant to P, CIP, gentamicin, and LEV with a resistance rate of 85.7% [30]. Finally, antibiotic resistance in bacteria that causes mastitis varies greatly between regions, highlighting the necessity of antibiotic susceptibility tests and frequent surveillance of antibiotic susceptibilities in bacteria that cause mastitis. In Gram-negative bacteria, resistance to β-lactam antibiotics is predominantly mediated by β-lactamases that hydrolyze the β-lactam ring and render the antibiotic inactive [27].

Antibiotic resistance increases morbidity, mortality, and the cost of treating infections, particularly those caused by ESBL-producing bacteria. The TEM, SHV, and CTX-M types are the most common ESBLs [12]. In all 80 strains in this study, *blaCTX* and *blaCMY-2* genes were not found, but *blaTEM* was the most often isolated gene, followed by *blaSHV* with 60% and 15% frequency rates, respectively. The occurrence results of *blaTEM*, *blaCTX-M*, and *blaSHV* were recorded previously [32], with frequency rates of 96.4%, 53.7%, and 34.5%, respectively.

A study conducted in Egypt [31] revealed that 17 (17%) of the 100 tested clinical strains of Enterobacteriaceae isolated from mastitic milk were ESBL positive. Genetic analysis of the 17 phenotypic ESBL-positive *E. coli* isolates showed that *blaCTX-M* was present in 41.2% (7 of 17) of ESBL-producing Enterobacteriaceae, whereas *blaTEM* and *blaSHV* were detected in 29.4% (5 of 17) and 11.8% (2 of 17), respectively. Eight isolates expressed the ESBL phenotype, but no *blaTEM*, *blaSHV*, or *blaCTX-M* was detected. In an investigation at Peninsular Malaysia, Kamaruzzaman *et al*. [34] reported that the predominant ESBL genotype detected in dairy cow’s milk and farm environment samples was a mix of *blaTEM* and *blaCTX-M* in 8 of 18 (44.4%) isolates. Four (22.2%) isolates produced the *blaCTX-M* gene, and 2 (11.1%) isolates produced the *blaTEM* gene. In a study in China [33], *blaCTX-M* was the predominant ESBL gene detected in 28 (77.78%) isolates. Twenty (55.56%) and 6 (16.67%) of the ESBL isolates carried the *blaTEM* and *blaSHV* genes, respectively. In terms of resistance genes identified, bacterial population resistance patterns vary among countries.

Samples were selected for serological examination that showed complete resistance to antibiotics, and the resistance genes *blaTEM* and *blaSHV* confirmed that ESBL-producing *E. coli* isolates were of the following serotypes: O26:k60, O125:k70, and O25:k11. Studies examined serotyping of ESBL-producing *E. coli*. Ahmed *et al*. [30] reported that ESBL-producing *E. coli* isolates in Egypt were determined by O-serotyping as O8, O86, and O157. Batabyal *et al*. [38] illustrated that different serotypes, such as O11, O20, O22, O34, O35, O128, and O149, were identified from ESBL-producing *E. coli* isolates from the collected samples. Another study in Austria [39] showed that a wide range of *E. coli* serogroups was involved in bovine mastitis carrying different antibiotic resistance genes. Twelve different O serogroups (O25, O26, O55, O78, O103, O114, O119, O125, O126, O128, O145, and O157) and *E. coli* serogroups, including those from this study, were found.

## Conclusion

This study revealed a significant prevalence of ESBL-producing *E. coli* in mastitic milk samples obtained from dairy cattle in four Egyptian provinces. It also reflected the prevalence of *blaTEM* and *blaSHV* as the most common ESBL genotypes in dairy cows. Furthermore, cattle and milk can act as potential ESBL gene reservoirs, allowing the propagation and maintenance of ESBL genes in humans, animals, and the environment to continue. Authorities should embrace the concept of One Health to keep the risk of new variants to a bare minimum. Individual dairy cows, rather than large-scale dairy cow production, could be considered a primary limitation to standing on the level of antimicrobial-resistant gene dispersion in Egypt. The study’s future scope is to develop a quick, field-applicable diagnostic test for antimicrobial-resistant genes.

## Authors’ Contributions

SSE, RHE, MKE: Conceptualization. SSE, RHE, MKE, AMA: Analysis. SSE, RHE, HAAA: Funding acquisition. AMA, HAAA: Investigation. MKE, SSE, RHE: Methodology. SSE, HAA: Resources. MKE, RHE: Supervision. MKE, SSE, AME: Visualization. SSE, MKE, RHE: Writing - original draft. MKE, HAAA, RHE, AMA, SSE: Writing - review and editing. All authors read and approved the final manuscript.
